# Cryptosporidiosis burden among children younger than 5 years with diarrhea attending Banadir hospital in Mogadishu, Somalia

**DOI:** 10.1016/j.ijregi.2026.100942

**Published:** 2026-07-06

**Authors:** Mohamed Adam Mahamud, Umeyma Ahmed Barre, Faadumo Abdirahman Ali, Abdikani Omar Salah, Ahmed Mahad Sheikh Mohamed, Jamal Hassan Mohamoud, Mohamed Hussein Adam, Hodo Aideed Asowe, Abdkerem Abdulahi Abdi, Bashiru Garba, Abdelhakam G. Tamomh, Mohamed Mustaf Ahmed

**Affiliations:** 1 Department of Microbiology and Laboratory Sciences, Faculty of Medicine and Health Sciences, SIMAD University, Mogadishu, Somalia; 2 SIMAD Institute for Global Health, SIMAD University, Mogadishu, Somalia; 3 Department of Public Health, Faculty of Medicine and Health Sciences, SIMAD University, Mogadishu, Somalia; 4 Department of Nursing and Midwifery Science, Faculty of Medicine and Health Sciences, SIMAD University, Mogadishu, Somalia; 5 Department of Parasitology and Medical Entomology, Faculty of Medical Laboratory Sciences, University of El Imam El Mahdi, Kosti, Sudan

**Keywords:** Cryptosporidiosis, Diarrhea, Children under five, Prevalence, Risk factors, Somalia

## Abstract

**Objectives:**

Diarrheal diseases remain a major cause of morbidity and mortality among children aged <5 years, particularly in low-resource settings. *Cryptosporidium* infection is an important contributor to pediatric diarrhea; however, no published data exist on its burden among children aged <5 years in Somalia. This study aimed to determine the prevalence of cryptosporidiosis among children with diarrhea attending Banadir hospital in Mogadishu, Somalia, and to assess its association with sociodemographic, exposure, and clinical factors.

**Methods:**

A hospital-based cross-sectional study was conducted among 315 children aged <5 years presenting with diarrhea. Sociodemographic and clinical data were collected using structured questionnaires. Stool samples were obtained and examined for *Cryptosporidium* spp. using standard laboratory diagnostic procedures. Statistical analyses were performed to assess associations between infection status and potential risk factors, with odds ratios and significance testing applied where appropriate.

**Results:**

*Cryptosporidium* infection was detected in 65 of 315 children (20.6%) presenting with diarrhea. Abdominal swelling was significantly associated with infection (*P =* 0.003). Children who had never been breastfed were significantly more likely to be infected, with 4.47-fold increased odds compared with children who had been breastfed. Sociodemographic variables, including age, sex, and parental education level, were not significantly associated with infection.

**Conclusion:**

Cryptosporidiosis represents an important contributor to diarrheal illness among children aged <5 years in Mogadishu. Routine screening for *Cryptosporidium* infection in diarrheal cases and promotion of breastfeeding may help identify cases and reduce infection risk. Further research is needed to better understand transmission patterns and the broader health impacts of cryptosporidiosis in this population.

## Highlights

• First report of pediatric cryptosporidiosis among diarrheal cases in Mogadishu.
• Hospital-based cross-sectional study of 315 children aged <5 years with diarrhea.
• Cryptosporidium prevalence was 20.6% among diarrheal cases in children aged <5 years.
• Cryptosporidium positivity was associated with abdominal swelling (*P =* 0.003).
• Never-breastfed children had a higher positivity proportion.
• The findings support routine testing and targeted prevention in high-burden settings.

## Introduction

Diarrheal disease remains a major cause of illness and preventable death in early childhood, with nearly 1.7 billion episodes of childhood diarrhea globally each year and about 443,832 deaths among children aged <5 years annually [1]. This burden is concentrated in low-resource settings, where unsafe water, inadequate sanitation, and delayed or incomplete treatment amplify dehydration risk and prolong infectious shedding, making timely etiologic identification and targeted prevention particularly important in high-transmission communities. Among the parasitic causes of pediatric diarrhea, *Cryptosporidium* spp. is consistently ranked as a leading pathogen linked to severe disease, mortality, and longer-term harm. In 2016, acute cryptosporidiosis was estimated to cause more than 48,000 deaths among children aged <5 years and more than 4.2 million disability-adjusted life years, with additional evidence linking infection to growth faltering that can compound vulnerability to future illness [2]

Regional syntheses emphasize that the burden is disproportionately borne by Africa, with estimates suggesting roughly 75% of cryptosporidiosis-associated diarrheal episodes and about 88% of related deaths among children aged <5 years occur on the continent [2]. In sub-Saharan Africa specifically, modeling grounded in multicountry case-control evidence has estimated approximately 2.9 million *Cryptosporidium*-attributable diarrheal episodes annually among children younger than 24 months, highlighting intense transmission during infancy and the second year of life [3]. Even within sub-Saharan Africa, the reported facility-based prevalence of diarrhea among children aged <5 years varies widely across sites and diagnostic methods, complicating local planning and clinical decision-making. For example, an outpatient study in Blantyre, Malawi, reported a *Cryptosporidium* prevalence of 9.1% among children aged <5 years with diarrhea using rapid diagnostic testing [4], whereas a large facility-based study in eastern Ethiopia found a prevalence of 15.2% using light-emitting diode (LED) fluorescence microscopy and identified modifiable exposures, such as non-exclusive breastfeeding and poor hygiene practices, as significant correlates [5]. Clinically, cryptosporidiosis can resemble other causes of acute watery diarrhea, and Africa-focused multicountry analyses have shown that age, study site, and season can influence presentation and risk, reinforcing the need for setting-specific data to support triage, testing decisions, and prevention targeting [6,7].

In Somalia, the urgency for local evidence is heightened by severe child-survival and Water, Sanitation, and Hygiene (WASH) constraints documented in authoritative United Nations sources, including a mortality rate for children aged <5 years of 104 deaths per 1000 live births, oral rehydration salts (ORS) coverage of only 16% among children aged <5 years diarrhea cases, safely managed sanitation coverage of 16%, and 75% of the population using at least basic drinking-water services [8]. However, contemporary, laboratory-linked estimates of cryptosporidiosis among children aged <5 years with diarrhea in Mogadishu remain limited, and the literature rarely integrates key exposure variables (such as breastfeeding status, household and animal contact, and sanitation-related proxies) with symptom patterns in a way that is immediately actionable for clinicians and public health planners. This gap matters because the study’s tables explicitly capture sociodemographic and exposure factors (including age group, residence, breastfeeding status, and pet contact) alongside symptom profiles (including abdominal pain, vomiting, and fever) within a major referral context where Banadir hospital has 700 beds, enabling evidence that can inform locally tailored prevention and diagnostic suspicion [9]. Therefore, the primary objectives of this study were to estimate the prevalence of laboratory-confirmed cryptosporidiosis among children aged <5 years presenting with diarrhea at Banadir hospital in Mogadishu, Somalia, and to assess associations between cryptosporidiosis and the sociodemographic, exposure, and clinical variables in this patient population.

## Methods

### Study area

The research took place at the pediatric department of Banadir hospital in Mogadishu, Somalia. Banadir hospital, one of the largest healthcare facilities in Somalia, offers comprehensive medical services with a particular focus on maternal and child health. It has a capacity of over 700 beds and is equipped with various departments, including pediatrics, internal medicine, surgery, gynecology, and infectious diseases, serving as a referral canter for complex cases. The hospital provides advanced diagnostic and treatment services, including intestinal parasites through laboratory diagnosis, making it a crucial site for this study.

### Study design

A hospital-based cross-sectional study was conducted to determine the prevalence of cryptosporidiosis among children aged <5 years with diarrhea attending Banadir hospital in Mogadishu, Somalia.

### Target population

The study was focus on children aged <5 years with diarrhea attending Banadir hospital in Mogadishu, Somalia.

### Inclusion and exclusion criteria

#### Inclusion criteria

The study was restricted to children who were aged ≤5 years. They involved those that had diarrhea and parents/guardians agreed for their children to be part of the study after explaining to them in English and Somali the objectives of our study.

#### Exclusion criteria

We excluded any participants who did not consent to be a part of the study and children without diarrhea.

### Sample size determination

Participants were selected using convenience sampling among children aged <5 years with diarrhea attending Banadir hospital during the study period. Given the lack of prior prevalence estimates in this region, sample size planning was based on conventional parameters for prevalence studies. Ultimately, 315 children were enrolled due to time and operational constraints; this sample size was considered adequate to estimate prevalence and explore key associations within this hospital-based population.

### Data collection techniques

A questionnaire was used as the data collection method. The questionnaire for this study is divided into two main sections: demographic information and clinical information.

### Questionnaire survey

A well-structured questionnaire was developed from a similar study conducted among children aged <5 years and their mothers in Somalia and used for data collection. The questionnaire comprised of two sections: sociodemographic characteristics of the participants (age, sex, residential area) and clinical symptoms (cryptosporidiosis screening history). We then also collected the stool sample.

### Sample collection and laboratory processing

Parents or guardians of children with gastrointestinal symptoms with diarrhea were provided with labeled stool containers to collect one stool sample at the time of collection, with instructions on how to gather an appropriate amount of stool and send it to the laboratory promptly. Appropriate gloves were worn during sample handling, and the stool samples were checked for quantity and physical appearance, with labels matched to the corresponding questionnaires. Fresh samples were stored at −20°C in the clinical laboratory’s freezer for later analysis. The samples were then allowed to reach room temperature, and fecal smears were prepared on microscope slides using wooden stick applicators, left to air dry. For staining, the slides were fixed in absolute methanol for 3 minutes, followed by a 3-minute application of strong carbol fuchsin stain, rinsed in tap water, and decolorized with 1% hydrochloric acid alcohol for 30 seconds, before being rinsed again. The slides were counterstained with 1% methylene blue for 3 minutes, rinsed, and air dried. The stained slides were examined microscopically using 40× and 100× magnification to detect oocysts. Oocysts, typically round to oval, 4-6 μm in size, were evaluated for acid-fast staining.

### Data analysis

Statistical software was used to evaluate the data. The demographic information was compiled using descriptive statistics, and the prevalence of cryptosporidiosis was determined. To find any correlations between the presence of cryptosporidiosis and demographic characteristics (age, sex, and living conditions), binary logistic regression was deployed. A statistically significant *P*-value is <0.05. For ease of interpretation, the results are displayed in the Tables.

### Ethical consideration

Ethical approval for the study was obtained from SIMAD University before commencing the research. The research proposal was reviewed and approved by the SIMAD University Research and Ethics Committee (approval reference: 2025/SU-IRB/FMHS/P0030). Both written and verbal consent were obtained from the parents of all enrolled children prior to their participation in the study.

## Results

### Sociodemographic characteristics of participants

A total of 315 respondents participated in the study. Among them, 52.1% were girls and 47.9% were boys (Table 1). Most of the children were <2 years of age (54.3%), while the remaining 45.7% were between 2 and 5 years old. Most participants resided in urban areas (85.7%) and all reported access to both tap water and latrine facilities. Regarding parental education, 51.7% of mothers and 46.7% of fathers were illiterate, with only 15.6% and 27.6%, respectively, having completed high school or higher. In terms of economic status, over half of the families (54.3%) had low-income, and 56.5% of guardians were unemployed. Additionally, 44.8% of the children had contact with domestic animals, while 27.3% had ever been breastfed. In this study, ever breastfed refers to whether the child had ever been breastfed before enrollment, not current breastfeeding status.

**Table 1:** Sociodemographic characteristics of respondents (N = 315).

Variables	Frequency	Percent (%)
**Sex**		
Male	151	47.9
Female	164	52.1
**Age**		
<2 years	171	54.3
2-5 years	144	45.7
**Location of residence**		
Urban	270	85.7
Rural	45	14.3
**Mother education level**		
Illiterate	163	51.7
Primary school	103	32.7
High school and above	49	15.6
**Father education level**		
Illiterate	147	46.7
Primary school	81	25.7
High school and above	87	27.6
**Contact with pet**		
Yes	141	44.8
No	174	55.2
**Ever breastfed**		
Yes	86	27.3
No	229	72.7
**Availability of latrine**		
Yes	315	100.0
**Family income level**		
Low	171	54.3
Middle	112	35.6
High	32	10.2
**Occupational status of child’s guardian**		
Employed	137	43.5
Unemployed	178	56.5

### Clinical symptoms among respondents

Among the 315 respondents, clinical symptom assessment revealed that abdominal pain was the most commonly reported symptom, affecting 88.9% of the children, followed by fever in 42.2% and nausea with vomiting in 30.2%. Abdominal swelling was reported by 6.0%, fatigue was present in 8.3%, and 16.8% of the children exhibited other symptoms (Table 2).

**Table 2:** Clinical symptoms among respondents (N = 315).

Variable	Category	Frequency	Percent (%)
**Abdominal pain**	Yes	280	88.9
	No	35	11.1
**Fever**	Yes	133	42.2
	No	182	57.8
**Abdominal swelling**	Yes	19	6.0
	No	296	94.0
**Nausea and vomiting**	Yes	95	30.2
	No	220	69.8
**Fatigue**	Yes	26	8.3
	No	289	91.7
**Others**	Yes	53	16.8
	No	262	83.2

### Bivariate logistic regression of sociodemographic and clinical characteristic associated with cryptosporidiosis

The bivariate and multivariate logistic regression analysis conducted on various sociodemographic and clinical factors revealed several associations with *Cryptosporidium* infection (Table 3). A notable finding was the lack of a significant relationship between most sociodemographic variables, such as age, sex, and residency, with *Cryptosporidium* infection, as indicated by high *P*-values (0.567, 0.776, and 0.887, respectively). However, certain clinical factors showed a stronger association. Notably, abdominal swelling was significantly associated with infection (*P =* 0.003), indicating a potential clinical marker for *Cryptosporidium*. Breastfeeding also showed a significant relationship, with those not breastfeeding more likely to test positive for *Cryptosporidium* (odds ratio = 4.468, *P =* 0.016), suggesting that breastfeeding may play a protective role. Despite these findings, other factors like fever, abdominal pain, and family income level did not show strong correlations with infection status. The data highlights that while some clinical symptoms and behaviors are linked to higher infection rates, sociodemographic factors like education level and family income showed no significant association.

**Table 3:** Bivariate logistic regression of sociodemographic and clinical characteristic associated with cryptosporidiosis (N = 315).

Variables	N (%)	*Cryptosporidium* infection		OR (95% CL)	*P*-value
		Negative	Positive		
**Age**					
<2 years	171 (54.3%)	147 (58.8%)	24 (36.9%)	1.237 (0.597-2.563)	0.567
2-5 years	144 (45.7%)	103 (41.2%)	41 (63.1)	1	
**Sex**					
Male	151 (47.9%)	124 (49.6%)	27 (41.5%)	1.107 (0.551-2.223)	0.776
Female	164 (52.1%)	126 (50.4%)	38 (58.5%)	1	
**Resident**					
Urban	270 (85.7%)	214 (85.6%)	56 (86.2%)	1.078 (0.383-3.032)	0.887
Rural	45 (14.3%)	36 (14.4%)	9 (13.8%)	1	
**Mother education**					
Illiterate	163 (51.7%)	116 (46.4%)	47 (72.3%)	0.517 (0.099-2.706)	0.435
Primary school	103 (32.7%)	92 (36.8%)	11 (16.9%)	0.602 (0.164-2.208)	0.444
High school and above	49 (15.6%)	42 (16.8%)	7 (10.8%)	1	
**Father education**					
Illiterate	147 (46.7%)	101 (40.4%)	46 (70.8%)	0.558 (0.105-2.958)	0.493
Primary school	81 (25.7%)	72 (28.8%)	9 (13.8%)	0.645 (0.183-2.273)	0.495
High school and above	87 (27.6%)	77 (30.8%)	10 (15.4%)	1	
**Contact with pet**					
Yes	141 (44.8%)	92 (36.8%)	49 (75.4%)	0.431 (0.179-1.039)	0.061
No	174 (55.25)	158 (63.2%)	16 (24.6%)	1	
**Breastfeeding**					
Yes	86 (27.3%)	82 (32.8%)	4 (6.2%)	4.468 (1.328-15.026)	0.016
No	229 (72.7%)	168 (67.2%)	61 (93.8%)	1	
**Family income level**					
Low	171 (54.3%)	122 (48.8%)	49 (75.4%)	0.820 (0.181-3.705)	0.797
Middle	112 (35.6%)	99 (39.6%)	13 (20.0%)	0.497 (0.110-2.235)	0.363
High	32 (10.2%)	29 (11.6%)	3 (4.6%)	1	
**Occupational status of child’s guardian**					
Employed	137 (43.35%)	127 (50.8%)	10 (15.4%)	2.106 (0.736-6.029)	0.165
Unemployed	178 (56.5%)	123 (49.2%)	55 (84.6%)	1	
**Abdominal pain**					
Yes	280 (88.9%)	219 (78.2%)	61 (21.8%)	0.522 (0.144-1.884)	0.321
No	35 (11.1%)	31 (88.6%)	4 (11.4%)	1	
**Fever**					
Yes	133 (42.2%)	115 (86.5%)	18 (13.5%)	1.929 (0.849-4.386)	0.117
No	182 (57.8%)	135(74.2%)	47 (25.8%)	1	
**Abdominal swelling**					
Yes	19 (6.0%)	4 (21.1%)	15 (78.9%)	0.120 (0.030-0.487)	0.003
No	296 (94.0%)	246 (83.1%)	50 (16.9%)	1	
**Nausea and vomiting**					
Yes	95 (30.2%)	72 (75.8%)	23 (24.2%)	1.482 (0.621-3.537)	0.376
No	220 (69.8%)	178 (80.9%)	42 (19.1%)	1	
**Fatigue**					
Yes	26 (8.35)	12 (46.2%)	14 (53.8%)	0.534 (0.152-1.939)	0.347
No	289 (91.7%)	238 (82.4%)	51 (17.6%)	1	
**Others**					
Yes	53 (16.8%)	23 (43.4%)	30 (56.6%)	0.311 (0.115-0.838)	0.21
No	262 (83.2%)	227 (86.6%)	35 (13.4%)	1	

Abbreviations: CL, confidence limit; OR, odds ratio.

## Discussion

This hospital-based analysis underscores the substantial role of *Cryptosporidium* as a significant contributor to diarrheal illness among children aged <5 years old presenting at Banadir hospital. Furthermore, our findings demonstrate that the infection profile is more closely associated with specific clinical presentations and feeding practices than with fundamental sociodemographic variables in this referral setting, suggesting an urgent need for targeted clinical awareness and intervention strategies. Additionally, our study is consistent with the broader evidence that diarrheal diseases remain a major cause of morbidity and mortality in children aged <5 years worldwide and in sub-Saharan Africa, and that *Cryptosporidium* is now recognized as a leading protozoal cause of pediatric diarrhea with important short-term and long-term consequences [2,10].

The detection of *Cryptosporidium* in stool should be interpreted cautiously, as pathogen positivity alone does not necessarily confirm it as the sole cause of diarrhea. In resource-limited settings, children are frequently exposed to multiple enteric pathogens, and asymptomatic carriage or prolonged shedding may occur even in the absence of diarrheal symptoms [10,11]. In addition, mixed enteric infections are common, and the presence of more than one pathogen can make it difficult to attribute diarrheal illness to a single organism [12] Therefore, the findings of this study should be interpreted as showing an association between *Cryptosporidium* detection and diarrheal illness, rather than definitive evidence that *Cryptosporidium* was the only causal pathogen in all cases. Future studies including asymptomatic controls, quantitative molecular testing, and broader enteropathogenic panels would help clarify the attributable role of *Cryptosporidium* and the contribution of co-infections.

Despite examining crucial sociodemographic factors such as age, sex, and residence, we found no statistically significant differences that separate infected from non-infected children. This observation may be attributed to the dense urban environment of Mogadishu where high population density and shared water resources create widespread exposure to enteric pathogens across various demographic strata. This saturation of exposure opportunities can diminish the distinctions that typically emerge in less densely populated areas, where sociodemographic factors might have more pronounced influences on infection risk. The implications of these findings align with the World Health Organization’s recognition of *Cryptosporidium’s* persistence in the environment and its propensity to contaminate even seemingly improved drinking-water sources. Therefore, reliance on basic household-level indicators of water quality may be insufficient for mitigating risk unless coupled with comprehensive assessments of water treatment practices, storage safety, and potential contamination events [2,10,13,14].

Concurrently, recent facility-based studies in the region have reported variable associations with caregiver education, hygiene practices, and household diarrhea exposure, suggesting that context and measurement detail matter; for example, an eastern Ethiopia study found multiple behavioral and household correlates that may not be captured by coarse infrastructure indicators alone [6,15].

Clinically, our study reveals a significant association between *Cryptosporidium* positivity and the presence of abdominal swelling. This symptom can be understood in the context of *Cryptosporidium’s* pathophysiology, which often involves intestinal inflammation, altered intestinal permeability, and subsequent malabsorption. These manifestations can exacerbate in children with repeated enteric infections or nutritional deficits, suggesting that abdominal distension may serve as a key clinical marker in diagnosing cryptococcal infections. Evidence from longitudinal studies supports that *Cryptosporidium*-associated diarrhea not only leads to acute symptoms but can also have profound long-term health implications, including growth deficits and overall developmental harms. Thus, recognizing non-specific gastrointestinal symptoms as potential indicators of significant infections is critical for timely intervention [2,11,[15], [16], [17]]. The feeding-related signal in our data, where children who were not breastfeeding were more likely to test positive, is consistent with established protective mechanisms of breastfeeding against enteric pathogens, including immune factors in human milk, reduced exposure to contaminated complementary foods, and improved mucosal resilience. Large-scale evidence underscores breastfeeding’s role in reducing infectious morbidity and mortality in early life [18,19].

Pathogen-focused work also suggests that breastfeeding can reduce protozoal diarrhea risk or severity in some settings, although effects may vary with background transmission intensity and feeding definitions, and not all cohort studies find strong differences once frequent exposure is established [19,20]. This nuance is important for interpretation in Mogadishu, where early, repeated exposure to enteropathogens is likely, and where breastfeeding promotion must be paired with safe complementary feeding and water handling to sustain protection beyond infancy, as also emphasized by diarrhea control frameworks centered on ORS plus zinc and on prevention through WASH improvements [21,22].

However, it is crucial to acknowledge several limitations in our study. The cross-sectional design inherently limits causal inferences and may over-represent severe cases, introducing a bias prevalent in hospital-based studies. Additionally, the reliance on modified acid-fast microscopy, while practical, may miss lower-intensity infections and is subject to operator variability, potentially affecting the accuracy of our prevalence estimates. The restriction of our sample to children reporting access to tap water and latrine facilities further constrains our ability to explore the effects of diverse WASH conditions, shifting focus toward service quality and household practices.

Despite these limitations, our findings advocate for a multipronged approach toward cryptosporidiosis management in Somalia. There is an urgent need to incorporate sensitive diagnostic tools, such as antigen-based assays or rapid tests, into routine clinical practice to elevate the level of suspicion for *Cryptosporidium* infections. Integrating cryptosporidiosis screening into established diarrheal management workflows, while maintaining core treatments like oral rehydration solutions and zinc supplementation, will enhance care for symptomatic children at high-burden facilities such as Banadir hospital.

Furthermore, expanding future studies to include multi-facility surveillance that captures seasonal trends, nutritional status, and molecular typing will be imperative in informing effective public health strategies, as regional burden estimates indicate that reducing cryptosporidiosis will yield significant health benefits beyond immediate diarrhea control, notably in preventing long-term growth and developmental impairments [13,21,23,24].

## CRediT authorship contribution statement

**Mohamed Adam Mahamud:** Conceptualization, Writing – original draft, Data curation, Methodology. **Umeyma Ahmed Barre:** Data curation, Writing – original draft. **Faadumo Abdirahman Ali:** Data curation, Investigation. **Abdikani Omar Salah:** Investigation. **Ahmed Mahad Sheikh Mohamed:** Methodology. **Jamal Hassan Mohamoud:** Conceptualization, Methodology. **Mohamed Hussein Adam:** Formal analysis. **Hodo Aideed Asowe:** Data curation, Formal analysis. **Abdkerem Abdulahi Abdi:** Data curation, Writing – review & editing. **Bashiru Garba:** Writing – review & editing. **Abdelhakam G. Tamomh:** Supervision. **Mohamed Mustaf Ahmed:** Conceptualization, Data curation, Writing – review & editing.

## Declaration of competing interest

The authors have no competing interests to declare.
